# Addressing decontaminated respirators: Some methods appear to damage mask integrity and protective function

**DOI:** 10.1017/ice.2020.332

**Published:** 2020-07-16

**Authors:** Richard E. Peltier, Jiayuan Wang, Brian L. Hollenbeck, Jennifer Lanza, Ryan M. Furtado, Jay Cyr, Richard T. Ellison, Kimiyoshi J. Kobayashi

**Affiliations:** 1 University of Massachusetts, Amherst, Massachusetts; 2 New England Baptist Hospital, Boston, Massachusetts; 3 UMass Memorial Medical Center, Worcester, Massachusetts

## Abstract

Decontamination of N95 respirators is being used by clinicians in the face of a global shortage of these devices. Some treatments for decontamination, such as some vaporized hydrogen peroxide methods or ultraviolet methods, had no impact on respiratory performance, while other treatments resulted in substantial damage to masks.

Frontline clinicians rely on the availability of personal protection equipment (PPE), such as N95 respirators, to reduce the risk of personal infection from exposure to respiratory droplets from infected individuals. The rise of a global coronavirus disease 2019 (COVID-19) pandemic has disrupted clinical supply chains that provide PPE, resulting in sustained shortages of N95 respirators, in part because of the widespread nature of the pandemic and the degree to which infected patients can present with varied symptoms.^1^ In an attempt to ameliorate this shortage, the US Food and Drug Administration issued an Emergency Use Authorization that allowed decontamination techniques for N95 respirators.^2^ This decision did not include mandate for further performance testing of respirators.

Several institutions have proposed the use of decontamination techniques to allow reuse of N95 respirators. Battelle has deployed a series of custom-designed vaporized hydrogen peroxide (vHP) sterilizers across the country.^3^ Others have proposed the use of different methods, such as gas plasma hydrogen peroxide (gpHP) or ultraviolet germicidal irradiation (UVGI) treatments. This methodology has eased the shortages of respirators, but an open question remains: Do respirators continue to protect a wearer after decontamination?

To assess post-decontamination efficacy, some institutions employ tests such as quantitative fit testing. Respirator performance is more complex than maintaining fit, and there remains a risk that fundamental aspects of respirators are degraded in a way that limits their performance, even though they retain acceptable fit parameters. Notably, N95^2^ is narrowly defined as a filtering material that is capable of filtering 95% of 300-nm particles and is largely agnostic to other particle types or sizes. Because respiratory droplets are expelled in a wide spectrum of sizes, typically ranging from 100 nm to 50 µm or more in diameter^4^ and because they persist in the environment for several minutes,^5^ respirators must be effective across a range of potential conditions to provide protection.

Given the global N95 shortages, clinicians face a choice: wearing a used, and potentially contaminated respirator, or wearing a respirator that was decontaminated through a process that may affect the integrity of the respirator. An urgent need exists to understand the quantitative effects on respirator filtration with the use of these techniques so that wearers remain protected.

## Methods

N95 respirators were obtained from hospitals actively using various decontamination techniques, and respirators were donned on a mannequin that was covered in a layer of soft closed-cell foam. The mannequin was installed in a 0.1-m^3^ exposure chamber and flooded with polydispersed combustion aerosol. For this study, almost all respirators were 3M 1860 or 1860S models (3M, St Paul, MN). Air was sampled through the mask at 85 L/min and alternated between chamber and mask-occluded sampling, consistent with a method in our prior work.^6^ Aerosol samples were delivered to a scanning mobility particle sizer (model 3225/3080, TSI, Minneapolis, MN), which characterized particle size distribution from 16.8 nm to 650 nm and provides much more detailed respirator performance information than standard filtration efficiency testing. Incense was burned in a separate combustion chamber and diluted ~50× prior to delivery to the exposure chamber. Using incense aerosol is a more protective assessment because respirators are less effective at capturing combustion aerosol^7^ compared to sodium chloride.

Respirator decontamination was performed off site using standard hospital processing protocols, and the quantity of masks was limited. The related data are summarized in Table 1.

**Table 1.** Decontamination Treatments Evaluated This Study, Including the Number of Respirators Evaluated and the Number of Treatments

Technique	Trade Name and Specific Cycles	No. of Respirators Tested	Cycle or Dwell Time (min)	Repeated Decontamination Treatments
Gas plasma HP	Sterrad 100S	1	55	1x
	Sterrad 100NX Express	5	28	1x, 3x, 5x
	Sterrad 100NX Standard	1	47	1x
Vaporized HP	Battelle CCDS	1	150	1x
	Bioquell	2	Varies	1x
	Steris V-Pro Max II	10	28	1x through 10x
Ultraviolet germicidal irradiation	Surfacide	10	10 (per side)	1x through 10x
10% diluted bleach	N/A	6	10	1x, 5x

Note. gpHP, gas-plasma hydrogen peroxide; vHP, vaporized hydrogen peroxide; UV, ultraviolet germicidal irradiation.

## Results

Respirators were characterized by filtration efficiency, which is a ratio of particles that are immobilized by a respirator relative to concentrations in the chamber. For each mask, this ratio is calculated for each of 107 different measured particle sizes.

**Fig. 1.**
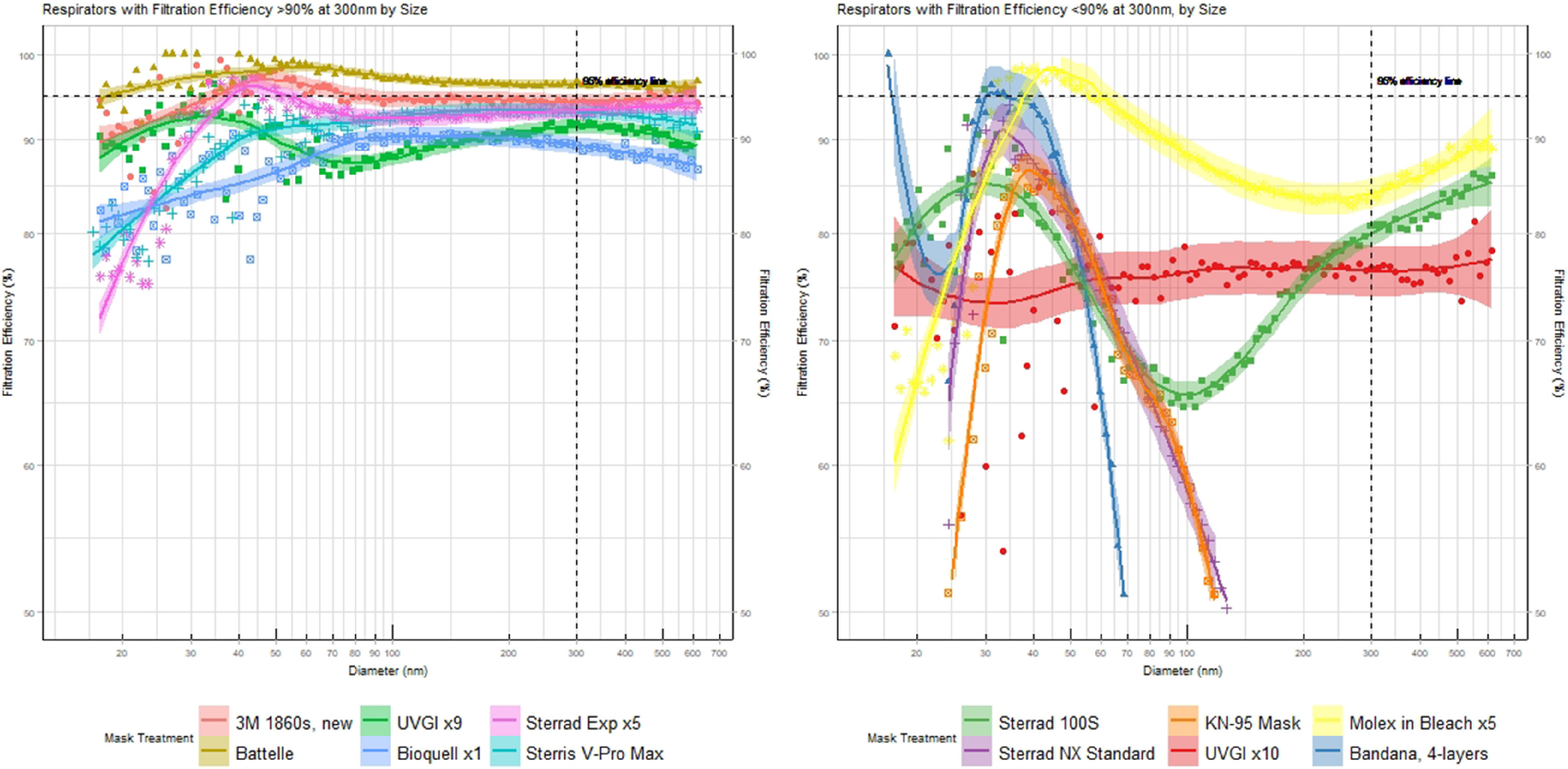
Filtration efficiency of N95 respirators across different decontamination treatments. (a) Plot on left includes masks where efficiency was >90% at 300 nm, and reflects a well-functioning mask. (b) Plot on right represents other decontamination methods where performance appears degraded. Dashed horizontal and vertical lines intersect at 300 nm and 95%, which is the basis for N95 designation. Shaded region indicates 95% confidence interval of the smoothed fit. In 1b, KN95, NX Standard, and Bandana efficiency are <50% at 150 nm, and it is not shown for clarity.

Figure 1 shows filtration efficiency across measured particle sizes across a spectrum from 16.8 nm to 650 nm. Experimental error was estimated at 5%; thus, when a particular mask was observed to filter 90% or more particles at 300 nm, it was deemed consistent with performance as a N95 respirator (Fig. 1a). Other respirators that do not meet this threshold were deemed inconsistent with this protection and are included in Figure 1b.

Respirators that were treated with vHP, or shorter decontamination cycles of gpHP, retained their original filtration capabilities. These included Battelle Critical Care Decontamination System (CCDS) and Bioquell (both processed just once), or Sterrad 100NX Express (processed up to 5 times). Steris V-Max Pro produced similar results; respirators maintained their filtration efficiency up to 10 treatments. Most ultraviolet treatments had minimal impact on respirator performance.

In contrast, gpHP treatment (by Sterrad 100S or Sterrad 100NX standard) degrades respirator filtration performance, with substantially decreased collection efficiencies across the entire size distribution. UVGI appears to degrade respirator performance after 9 repeated cycles.

For comparison, a KN95 mask (Dongguan Huagang), and a 4-ply polyester bandana are included in Figure 1. Neither were treated with any decontamination, with filtration efficiencies much worse than all masks in Figure 1a. We also evaluated performance of a N95 respirator that was immersed in a 0.5% bleach solution (Fig. 1b), and performance was also degraded.

## Discussion

Respirator performance can vary greatly. Respirators are surprisingly complex matrixes that can be deleteriously impacted by external forces, such as damaging interaction with strongly oxidizing environments. Although the intent of decontamination is to furnish a sanitized respirator for clinical reuse, some treatments result in respirators that offer less protection to wearers.

Although the evidence presented here are limited, there are some generalizable conclusion that can be drawn. Treatments that involve hydrogen peroxide and gas plasma at high concentrations (Sterrad 100NX Standard, concentration, ~90%), or long dwell times (Sterrad 100S) appear to induce damage to masks. Repeated UVGI processing appears to slowly diminish filtration efficiency, and this diminished efficacy reaches a level that warrants caution after ~9 repeated treatments. However, the Sterrad 100NX express cycle, which uses a lower concentration of gpHP and shorter time, has limited impact on respirator performance for up to 5 cycles. Our results are consistent with recent findings.^8,9^ Ou et al^10^ also tested UVGI with results consistent to those shown here, but their methods, which were quite similar to ours, provided more granular details on filtration performance than typical filtration efficiency testing.

In general, vHP appears to have less of a deleterious impact on respirator performance. Treatments involving vHP include Steris V-Pro Max (tested up to 10 repeated decontaminations) and Bioquell and Battelle CCDS (tested after only 1 decontamination cycle). We cannot draw any conclusions about whether repeated Bioquell or Battelle CCDS treatments were acceptable because we were unable to source sufficient respirators.

The results of this study do not address fit or general respirator integrity, which are also important for proper respirator function. For any respirator decontamination, respirator integrity should be assessed (including elastic function or corrosion on staples), and fit testing should be performed before use.

These results suggest that respirators continue to perform as expected when decontaminated, mainly by vHP, UVGI, or brief exposures to gpHP. Future studies could explore whether use of decontaminated respirators is linked to healthcare worker outbreaks as another potential indicator of respirator performance. Decontamination treatments need to be carefully considered by clinical staff, especially for decontamination methods not yet well understood, in the search for creative ways to provide sufficient respirators to clinical staff.
